# STAT5 promotes PD-L1 expression by facilitating histone lactylation to drive immunosuppression in acute myeloid leukemia

**DOI:** 10.1038/s41392-023-01605-2

**Published:** 2023-09-30

**Authors:** Ze-Wei Huang, Xue-Ning Zhang, Ling Zhang, Ling-Ling Liu, Jing-Wen Zhang, Yu-Xiang Sun, Jue-Qiong Xu, Quentin Liu, Zi-Jie Long

**Affiliations:** 1 Department of Hematology, The Third Affiliated Hospital, Sun Yat-sen University; Institute of Hematology, Sun Yat-sen University, Guangzhou, China; 2 Department of Nephrology, The Third Affiliated Hospital, Sun Yat-sen University, Guangzhou, China; 3 Sun Yat-sen University Cancer Center; State Key Laboratory of Oncology in South China, Sun Yat-sen University, Guangzhou, China

**Keywords:** Tumour immunology, Haematological cancer

## Abstract

Immunotherapy is a revolutionized therapeutic strategy for tumor treatment attributing to the rapid development of genomics and immunology, and immune checkpoint inhibitors have successfully achieved responses in numbers of tumor types, including hematopoietic malignancy. However, acute myeloid leukemia (AML) is a heterogeneous disease and there is still a lack of systematic demonstration to apply immunotherapy in AML based on PD-1/PD-L1 blockage. Thus, the identification of molecules that drive tumor immunosuppression and stratify patients according to the benefit from immune checkpoint inhibitors is urgently needed. Here, we reported that STAT5 was highly expressed in the AML cohort and activated the promoter of glycolytic genes to promote glycolysis in AML cells. As a result, the increased-lactate accumulation promoted E3BP nuclear translocation and facilitated histone lactylation, ultimately inducing PD-L1 transcription. Immune checkpoint inhibitor could block the interaction of PD-1/PD-L1 and reactive CD8^+^ T cells in the microenvironment when co-culture with STAT5 constitutively activated AML cells. Clinically, lactate accumulation in bone marrow was positively correlated with STAT5 as well as PD-L1 expression in newly diagnosed AML patients. Therefore, we have illustrated a STAT5-lactate-PD-L1 network in AML progression, which demonstrates that AML patients with STAT5 induced-exuberant glycolysis and lactate accumulation may be benefited from PD-1/PD-L-1-based immunotherapy.

## Introduction

AML is a heterogeneous and immunosuppressive malignancy that is characterized by the expansion of myeloblasts with differentiation blockage.^1^ It accounts for more than eighty thousand deaths globally per annum, and the number of mortality will be more than double by the next two decades.^2^ 10–40% of newly diagnosed patients fail to achieve completed remission after initial treatment, and a portion of patients experience relapse even they have obtained completed remission.^3,4^ Thus, more effective therapies are urgently needed for AML treatment.

The immune system is the guardian of organismal integrity, which protects humans from certain diseases, such as infection and tumor.^5^ However, tumors have evolved multiple biological modes to escape immune surveillance, such as blockage of antigen presentation and recruitment of immunosuppressive cells.^6–8^ Programmed cell death protein 1/programmed cell death-ligand 1 (PD-1/PD-L1) is one of the most pivotal immune checkpoint axes that restrains the hyperactivation of immune cells and prevents autoimmune diseases.^9^ Meanwhile, the upregulation of PD-1/PD-L1 negatively regulates lymphocyte activation and manipulates an immunosuppressive microenvironment in tumors.^10^ The evasion of leukemic cells from immune surveillance leads to the failure in AML treatment.^11^ Previous study has revealed that PD-L1 and/or PD-L2 can be detected in AML patients with poor prognosis,^12^ and PD-1 expression is also significantly elevated in CD8^+^ T cells of patients with newly diagnosed and relapsed AML compared with healthy donors,^13^ implicating that immune checkpoint proteins would be applied as therapeutic targets in AML. Nowadays, immune checkpoint inhibitors have been underway in myeloid malignancy. In a phase 2 trial, relapsed/refractory AML patients achieve an encouraging response rate and overall survival after treatment with PD-1 inhibitor and azacytidine.^14^ Limited activity has been also noticed in clinical trials in which single-agent benefits are restricted to patients after allogeneic hematopoietic stem cell transplantation.^15^ Indeed, a considerable portion of patients fail to experience a response to the immunotherapy in AML.^16,17^ Hence, elucidating the underlying mechanism driving PD-L1 expression offers a rationale for the prognostic prediction of immunotherapy in AML.

STAT5 is a transcription factor that is reported to be essential for multilineage hematolymphoid development.^18^ The recruitment of STAT5 regulates gene expression by phosphorylation, dimerization and translocation to the nucleus followed by binding to the DNA sequence.^19^ Constitutive activation of STAT5 has been noticed in hematopoietic malignancy and is associated with resistance to tyrosine kinase inhibitors.^20,21^ The direct evidence that STAT5 acts as an oncogene is mice receiving constitutively activated STAT5 (S711F)-transduced bone marrow develop to multilineage leukemia.^22^ It is widely established that multiple metabolic processes are associated with STAT5 expression. The JAK/STAT pathway, especially STAT5, has been proven to participate in glucose metabolism by promoting the activation of AKT^23^ and the expression of HIF-2α.^24^ In leukemic T cells, STAT5 displays unique DNA binding activity and shifts the cellular metabolism to aerobic glycolysis.^25^ In KG1 cells transduced with constitutively active STAT5, the expression of mitochondrial genome-encoded MT-ATP6 and MT-CYB, as well as mitochondrial mass are significantly increased.^26^ Previous studies reveal that aerobic glycolysis promotes PD-L1 expression and leads to tumor immune evasion while blocking the engagement between PD-1 and PD-L1 permits T cell glycolysis and IFN-γ production.^27,28^ Meanwhile, tumor cell-derived lactate by glycolysis repressed cytotoxic T lymphocytes by a decrease in proliferation and cytokine production, suggesting targeting lactate metabolism is a promising approach to enhance tumor immunogenicity.^29^ However, whether STAT5 can trigger lactate production by glycolysis to drive immunosuppression in AML needs to be elucidated.

Post-translational modifications (PTM) of histone comprise part of epigenetic regulatory mechanism, such as acetylation, methylation, phosphorylation and palmitoylation. Recently, glycolysis-derived lactate has been identified to be a substrate for histone lactylation which is catalyzed by p300^30^ and removed by class I histone deacetylases (HDAC1-3).^31^ Lactylation has been proven to be associated with various physiological or pathological processes. Histone lactylation of H3K18 at the loci of pluripotency genes facilitates cellular reprogramming in somatic cell differentiation.^32^ In lung myofibroblasts, histone lactylation on the promoters of the profibrotic genes increases their expression in macrophage.^33^ Histone lactylation also participates in immune regulation. During M1 macrophage polarization, increased H3K18 lactylation significantly induces the expression of homeostatic genes involved in wound healing, such as Arg1.^30^ Besides, the transition of macrophage from inflammatory to reparatory status is regulated by BCAP-promoting histone lactylation.^34^ H3K18 lactylation also leads to immunosuppression by inducing METTL3 expression, which mediates m^6^A modification on JAK1 mRNA in tumor-infiltrating myeloid cells and subsequently phosphorylates STAT3,^35^ implying that targeting protein lactylation may be also a new approach for tumor therapy.

In this study, we reported that glycolysis was upregulated by STAT5 in AML. Importantly, the accumulation of lactate induced by STAT5 facilitated E3-binding protein (E3BP) nuclear translocation, increased the lactylation level of PD-L1 promoter and subsequently induced PD-L1 transcription in leukemic cells. Blockage of PD-1/PD-L1 could reactivate CD8^+^ T cells co-cultured with STAT5 high-expressed AML cells. Our finding demonstrates the molecular basis for lactylation-regulated PD-L1 expression and offers the potential application of immunotherapy for the clinical treatment in AML.

## Results

### STAT5 attenuates immune status by interfering with T cell function in AML patients

**Fig. 1:**
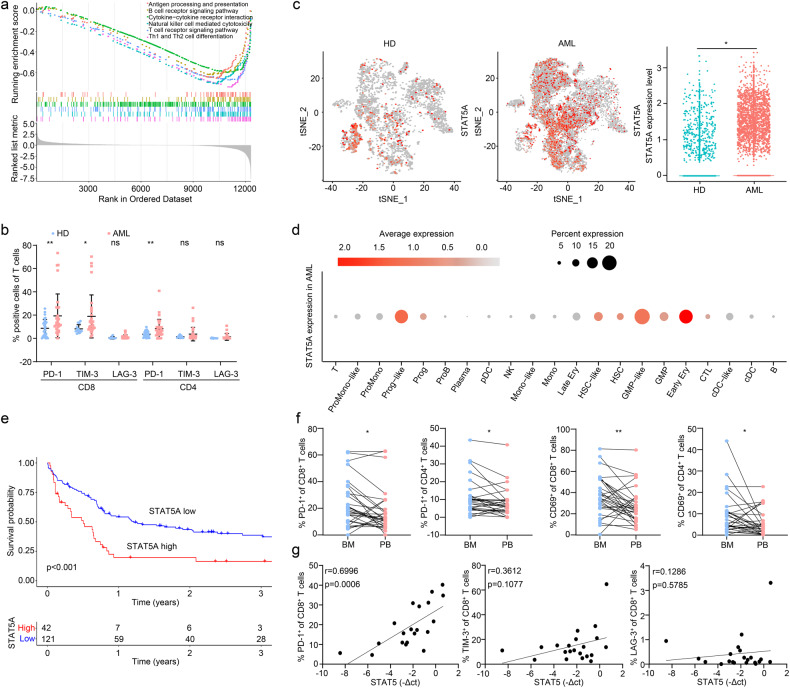
STAT5 predicts the poor prognosis accompanied with T cell dysregulation. **a** GSEA was performed in BM of 7 AML patients vs. 10 healthy donors derived from GSE9476. **b** PBMCs were isolated from AML patients and healthy donors (HD). The exhaustion of CD4^+^ and CD8^+^ T cells was determined by the expression of PD-1 (28 healthy donors vs. 32 AML patients), TIM-3 (13 healthy donors vs. 32 AML patients) or LAG-3 (13 healthy donors vs. 32 AML patients) on cell surface respectively. **c** t-SNE visualization of STAT5 expression in 16 AML patients and 4 healthy donors derived from GSE116256. STAT5 expression in each cell was normalized by Z-score and illustrated in a dot plot, and further analyzed between healthy donors and AML patients. **d** Dot plot visualization of STAT5A expression in 15685 cells of 16 AML patients derived from GSE116256. **e** Survival data of 163 AML patients derived from GSE12417 was analyzed. The study population was divided by the expression of STAT5A gene. **f** PBMCs and BMMCs were isolated from AML patients. The expression of PD-1 and CD69 (31 BM vs. 31 PB) was determined. **g** The expression of STAT5 and the markers of CD8^+^ T cells including PD-1 (Supplementary Table S1; No. 1-20), TIM-3 (Supplementary Table S1; No. 1-21) and LAG-3 (Supplementary Table S1; No. 1-21) were detected in AML BMMCs, followed by the Person’ correlation analysis. Data were represented as mean ± SD. *p < 0.05, **p < 0.01, ns not significant

Dysregulated immune regulation has been recognized to be a key pathogenic driver of AML. To gain insights into the immune heterogeneity of normal and malignant hematopoiesis, we performed GSEA between healthy donors and AML patients by employing the GSE9476 dataset. The result showed that multiple immune-related pathways were downregulated in AML patients (Fig. 1a). Since T cells encounter exhaustion due to immunosuppression during tumor progression, we next divided T cells into three subgroups based on the specific markers and found that cytotoxic T lymphocytes (CTL) tended to decrease in AML patients compared to healthy donors derived from GSE116256 (Supplementary Fig. 1a, b). By verifying the expression of T cell markers on peripheral blood mononuclear cells (PBMCs) of AML patients and healthy donors, we observed that the expression of PD-1 and TIM-3 was distinctly upregulated on CD8^+^ T cells of AML patients (Fig. 1b). These results suggested that the immune status of T cells in AML patients was strongly dysregulated.

Constitutive activation of STAT5 has been noticed in hematopoietic malignancy and is associated with leukemia initiation and development.^36^ We then investigated whether STAT5 contributed to the dysregulation of immune status in AML. The expression of STAT5 in AML and healthy donors was first analyzed by GSE116256. The results revealed that STAT5 was highly expressed in malignant cells of AML patients (Fig. 1c, d). Moreover, Survival analysis of 163 AML patients from GSE12417 showed high STAT5 expression was significantly associated with short overall survival (Fig. 1e). 16 AML patients derived from GSE116256 were further categorized into two groups based on the expression of STAT5 followed by an analysis of immune cell proportion. The result displayed that monocytes, promonocytes, conventional dendritic cells (cDCs) and B cells were increased, while proB cells, plasma cells and T cells were declined in STAT5 high-expressed AML patients (Supplementary Fig. 1c). We next compared the difference in T cell function between bone marrow mononuclear cells (BMMCs) and PBMCs of AML. Despite the comparable expression of TIM-3 and LAG-3 in BMMCs and PBMCs (Supplementary Fig. 1d), PD-1 and CD69 expression were increased in BMMCs (Fig. 1f). Subsequently, we noticed that STAT5 expression was positively associated with PD-1 on CD8^+^ T cells of BMMCs (Fig. 1g). Thus, STAT5 could interfere with the function of T cells in AML patients.

### Constitutive activation of STAT5 promotes glycolysis in AML cells

**Fig. 2:**
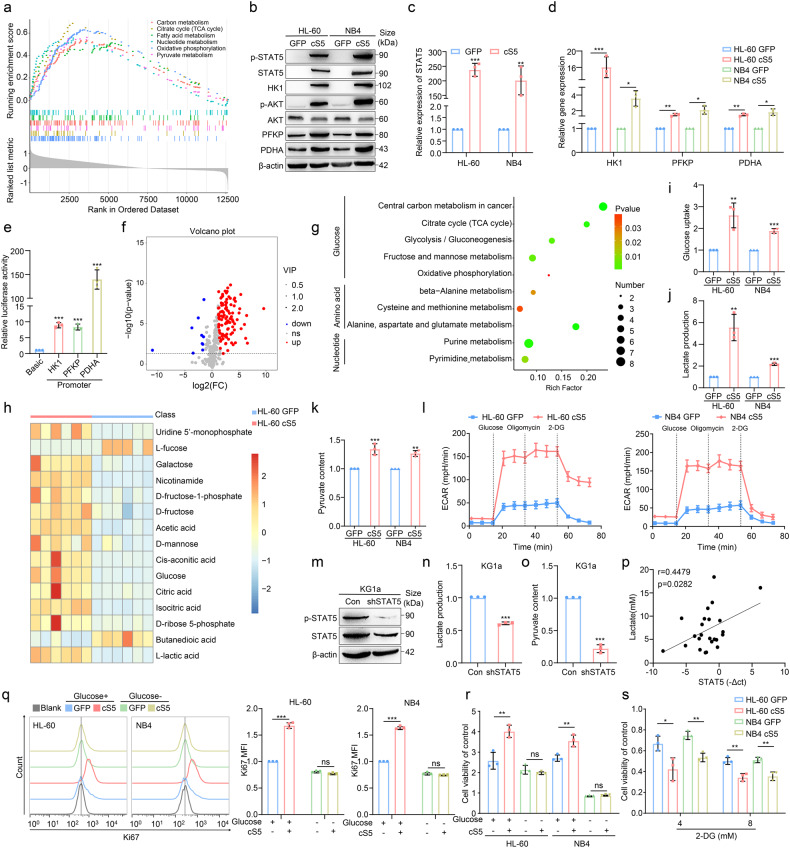
STAT5 is associated with glycolysis in AML cells. **a** 163 AML patients derived from GSE12417 were divided by quartiles of the expression of STAT5A and GSEA was performed. **b** Equal numbers of GFP (control) and cS5 AML cells were cultured for 24 h followed by western blot. **c**, **d** The expression of STAT5, HK1, PFKP and PDHA mRNA were detected by qPCR. **e** 293FT cells were transfected with indicated reporter constructs and pRL-TK followed by luciferase activity determination after culturing for 48 h. **f**, **g** GFP or cS5 HL-60 cells were collected for untargeted metabolomics analysis. Volcano plots and KEGG analysis were performed. **h** Heatmap visualization of the metabolites was shown in HL-60 cells. **i**–**l** Equal numbers of GFP and cS5 AML cells were cultured for 24 h, and glucometabolic index was determined by glucose uptake, lactate production, pyruvate content and ECAR. **m** The detection of STAT5 knock-down efficacy in KG1a was measured by western blot. **n**, **o** Equal numbers of control and STAT5 knock-down KG1a cells were cultured for 24 h followed by the determination of lactate production and pyruvate content. **p** Plasma from 24 AML patients (Supplementary Table S1; No. 1-24) were used for lactate detection. BMMCs from 24 AML patients were applied for the detection of STAT5 gene expression. Person’ correlation analysis was conducted for the correlation between STAT5 expression and lactate content in AML patients. **q** GFP and cS5 AML cells were cultured with or without glucose for 48 h followed by flow cytometry analysis of Ki67 to evaluate proliferative capacity. **r** Equal numbers of GFP and cS5 AML cells were cultured with or without glucose for 48 h. Cell viability was detected by CCK-8 assay. **s** Equal numbers of GFP and cS5 AML cells were cultured with or without 2-DG for 48 h. Cell viability was detected by CCK-8 assay. Data were represented as mean ± SD. *p < 0.05, **p < 0.01, ***p < 0.001, ns not significant

Since STAT5 is involved in controlling cellular metabolism,^23–26^ we continued to assess how STAT5 impacted on leukemic glycolysis by identifying the differentially expressed genes (DEGs) from GSE12417 and categorizing them into quartiles based on the expression of STAT5. GSEA revealed that metabolic pathways were upregulated in STAT5 high-expressed AML patients (Fig. 2a). We then constructed STAT5 constitutively activated (cS5) AML cells and detected the expression of glycolytic enzymes. STAT5 promoted glycolytic enzyme expression, including HK1, PFKP and PDHA. Meanwhile, phosphorylated AKT (p-AKT), an indicator for evaluating metabolic enhancement, was also elevated in cS5-overexpressed cells (Fig. 2b–d). Moreover, the luciferase reporter activity of glycolytic genes was potently induced by STAT5 (Fig. 2e). Hence, STAT5 could activate the promoter of HK1, PFKP and PDHA to promote their transcription followed by upregulation of glucose metabolism.

We then performed GC-MS untargeted metabolomics to investigate the metabolic products of cS5-overexpressed AML cells and found that 112 metabolites were altered (Fig. 2f). As presented in Fig. 2g, metabolic pathways including glucose, amino acid and nucleotide metabolism were changed in cS5-overexpressed AML cells by KEGG enrichment analysis. Notably, glucose, citric acid, isocitric acid, D-ribose 5-phosphate and L-lactic acid in glycolysis were increased in cS5-overexpressed AML cells (Fig. 2h). Consistently, cS5-overexpressed leukemic cells displayed a significant increase in glycolytic metabolism, as judged by enhanced glucose uptake (Fig. 2i), lactate production (Fig. 2j) and pyruvate content (Fig. 2k). Meanwhile, glycolysis stress test also indicated that extracellular acidification rate (ECAR) was increased in cS5-overexpressed AML cells (Fig. 2l). In addition, knock-down of STAT5 (Fig. 2m) decreased lactate production and pyruvate content (Fig. 2n, o). We further confirmed that the expression of STAT5 was positively correlated with lactate production in the bone marrow (BM) of AML patients (Fig. 2p). These results revealed that STAT5 could upregulate glycolysis in AML. We previously reported that STAT5 also significantly induced proliferation in AML cells.^37^ However, this effect was reinstated by removing glucose in the culture medium (Fig. 2q, r). Consistently, proliferation induced by cS5 could be reversed after exposing AML cells to 2-deoxy-d-glucose (2-DG) (Fig. 2s), suggesting that STAT5 enhanced leukemic growth mainly by increasing glycolysis.

### STAT5 mediates lactate-induced PD-L1 expression in AML cells

**Fig. 3:**
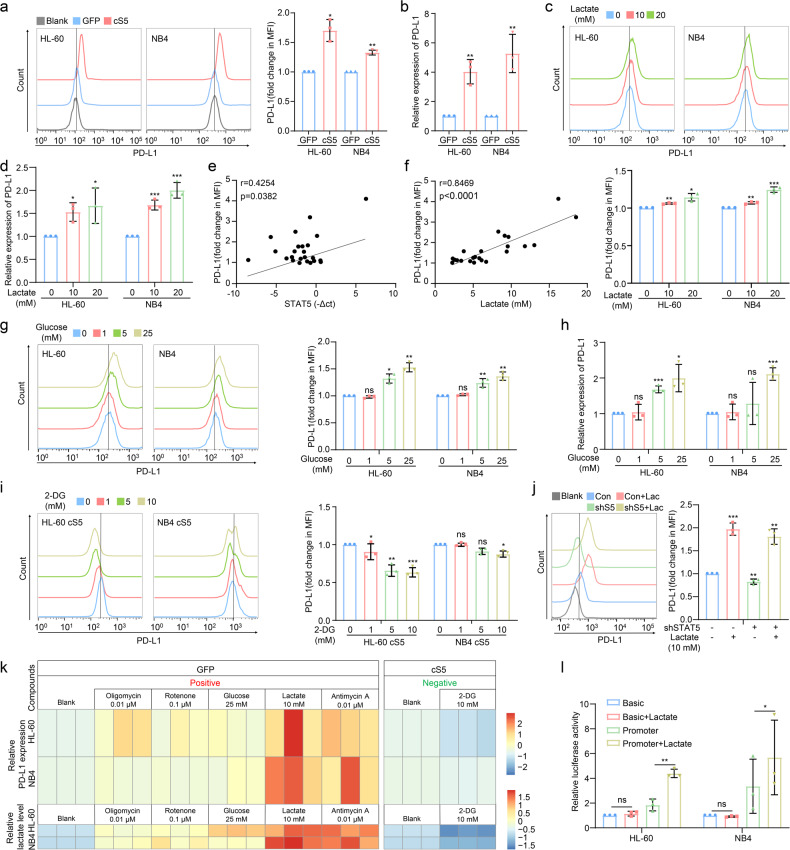
STAT5 regulates PD-L1 expression by increasing lactate production. **a**, **b** The protein expression of PD-L1 on cell surface or PD-L1 mRNA was detected by flow cytometry and qPCR respectively. **c**, **d** HL-60 and NB4 cells were exposed to lactate for 24 h, and subjected to flow cytometry and qPCR for PD-L1 expression. **e**, **f** The expression of PD-L1 in CD45^dim^SSC^dim^ BMMCs was detected by flow cytometry. Person’ correlation analysis was conducted to analyze the correlation between PD-L1 expression and lactate production or STAT5 expression. **g**, **h** HL-60 and NB4 cells were cultured in glucose-free culture medium and exposed to glucose for 24 h to determinate PD-L1 expression by flow cytometry and qPCR. **i** cS5 AML cells were cultured in glucose-free culture medium supplied with 25 mM glucose followed by exposure to 2-DG for 24 h to determinate PD-L1 expression by flow cytometry. **j** Control and STAT5 knock-down KG1a cells were exposed to lactate for 24 h, and then subjected to flow cytometry for PD-L1 expression. **k** HL-60 and NB4 cells were exposed to 0.01 μM oligomycin, 0.1 μM rotenone, 25 mM glucose, 10 mM lactate, or 0.01 μM antimycin A to increase lactate content, while cS5-overexpressed HL-60 and NB4 cells were exposed to 10 mM 2-DG to inhibit lactate production. After 24 h, intracellular lactate content and PD-L1 expression were detected. **l** HL-60 and NB4 cells were transiently transfected with pRL-TK, pGL3-basic or pGL3-PD-L1 promoter plasmid followed by luciferase activity determination. The medium without glucose was applied for 6 h to remove lactate, and then cells were treated with or without 10 mM lactate for another 24 h in the medium without glucose until luciferase activity determination. Data were represented as mean ± SD. *p < 0.05, **p < 0.01, ***p < 0.001, ns not significant

The enhancement of glycolysis in cS5-overexpressed AML cells led to the accumulation of lactate (Fig. 2j). In addition, tumor-derived lactate induces PD-L1 expression in some types of tumors.^38,39^ Thus, we explored the role of STAT5 in modulating the expression of PD-L1. PD-L1 expression was markedly upregulated by cS5 as determined by flow cytometric and qPCR analysis (Fig. 3a, b). In contrast, knock-down of STAT5 in KG1α reduced PD-L1 expression (Supplementary Fig. 2a, b). To address the biological relevance of lactate-induced PD-L1 expression in AML, lactate was directly supplied to AML cells and PD-L1 expression was induced (Fig. 3c, d and Supplementary Fig. 2c, d). This was supported by the result that PD-L1 expression was positively correlated with STAT5 expression or lactate content in the BM of AML patients (Fig. 3e, f). We further evaluated whether PD-L1 expression could be enhanced by glycolysis by exposing cells to the culture medium supplied with various doses of glucose. AML cells stimulated with high concentrations of glucose displayed a significant increase in glycolytic metabolism, as judged by the accumulation of intracellular and extracellular lactate (Supplementary Fig. 3a, b). As expected, flow cytometry and qPCR showed an increase in PD-L1 expression in glucose-stimulated AML cells (Fig. 3g, h). Conversely, when cS5-overexpressed AML cells were exposed to 2-DG, PD-L1 expression and lactate content were attenuated (Fig. 3i and Supplementary Fig. 3c, d). The reduction of PD-L1 in STAT5 knock-down AML cells could be restored by additional lactate (Fig. 3j). Furthermore, AML cells were exposed to different compounds which could switch oxidative phosphorylation to glycolysis. The results showed that PD-L1 expression was positively correlated with lactate production in AML cells (Fig. 3k). We then analyzed the effect of lactate on PD-L1 promoter activity using luciferase report assay. Importantly, PD-L1 promoter activity was distinctly enhanced after lactate exposure (Fig. 3l and Supplementary Fig. 3e). Therefore, lactate could stimulate PD-L1 promoter to initiate PD-L1 expression.

### Lactate activates PD-L1 gene expression through histone lactylation

**Fig. 4:**
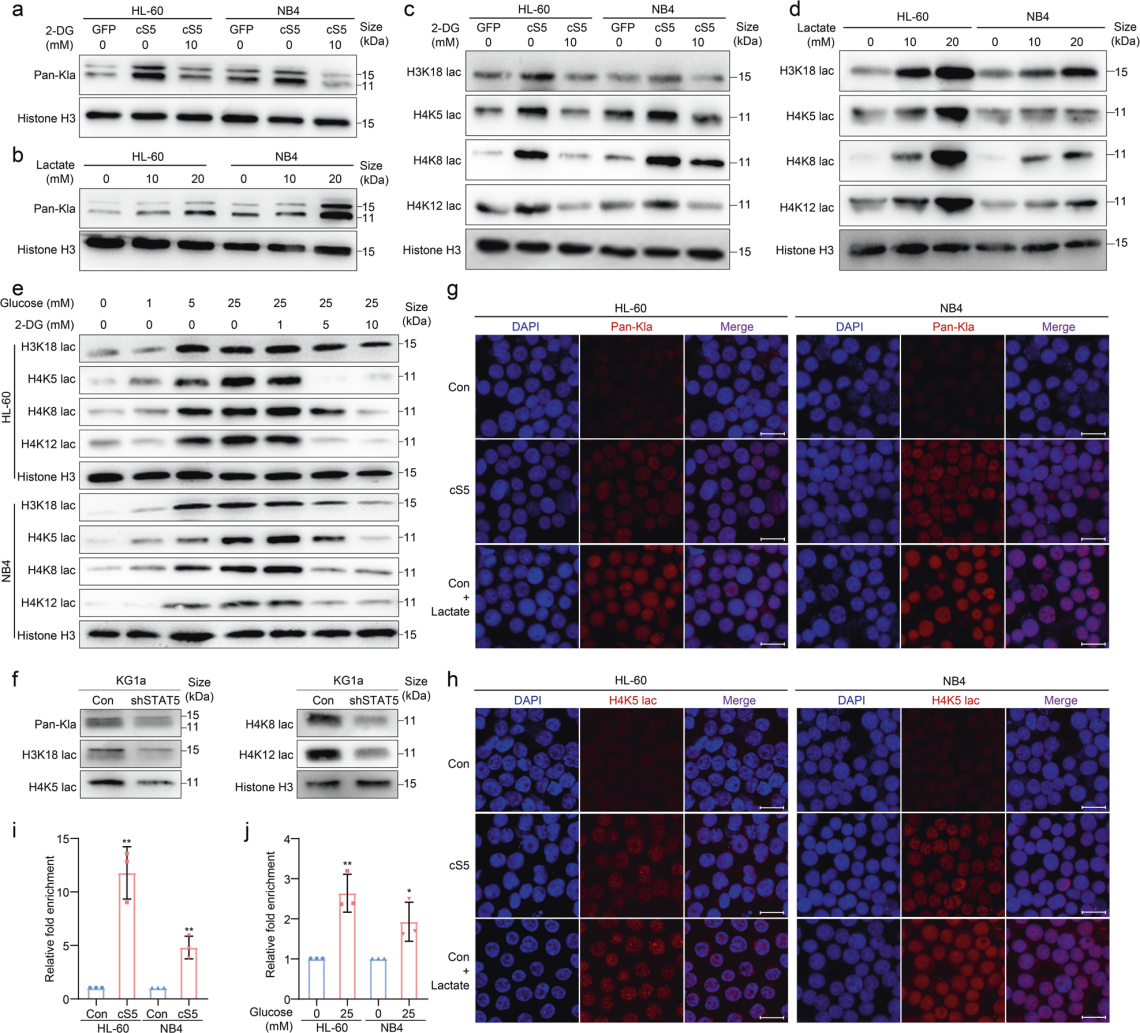
Lactate activates PD-L1 gene expression through histone lactylation. **a** Control and cS5-overexpressed cells were cultured with or without 2-DG for 24 h. Histone pan lactylation (Kla) was detected. **b** HL-60 and NB4 cells were cultured in a glucose-free culture medium for 6 h to remove intracellular lactate. Then glucose-free culture medium was supplied with various concentrations of lactate for 24 h and histone Kla was detected. **c** Cells were exposed to 2-DG for 24 h and site-specific histone lactylation was detected. **d** HL-60 and NB4 cells were cultured in a glucose-free culture medium for 6 h to remove intracellular lactate. Then glucose-free culture medium was supplied with various concentrations of lactate for 24 h and site-specific histone lactylation was detected. **e** HL-60 and NB4 cells were cultured in a glucose-free culture medium supplied with various concentrations of glucose or 2-DG for 24 h. Specific histone lactylation was detected. **f** Control and STAT5 knock-down KG1a were cultured for 24 h. Histone lactylation was detected. **g**, **h** Cells were exposed with or without lactate for 24 h. Histone Kla and H4K5 specific histone lactylation was detected by immunofluorescence. Scale bar = 20 μm. **i** Control and cS5-overexpressed cells were subjected to ChIP-qPCR using H4K5 lactylation antibody. **j** HL-60 and NB4 cells were cultured in glucose-free culture medium supplied with or without glucose for 24 h and subjected to ChIP-qPCR using H4K5 lactylation antibody. Data were represented as mean ± SD. *p < 0.05, **p < 0.01

As STAT5 could improve lactate production in AML (Fig. 2j) and lactylation is a novel post-translational modification mediated by lactate,^30^ we further studied whether lactate could drive lactylation-mediated PD-L1 expression. Indeed, STAT5 could increase pan lactylation (Kla) of histone in AML cells while 2-DG showed the contrary (Fig. 4a). We then cultured AML cells with additional lactate and noted that histone Kla was significantly induced (Fig. 4b). Next, we evaluated the lactylation level of specific residue on histone between control and cS5-overexpressed AML cells. STAT5 induced the lactylation level of H3K18, H4K5, H4K8 and H4K12 (Fig. 4c). Additional lactate in culture medium significantly induced histone lactylation of specific residues (Fig. 4d). Consistently, modification of lactate production by exposing AML cells to various concentrations of glucose or 2-DG could alter the lactylation, respectively (Fig. 4e). In addition, attenuated histone lactylation was observed in STAT5 knock-down AML cells (Fig. 4f). Meanwhile, the immunofluorescence analysis also supported the above results (Fig. 4g, h). Lactate-derived lactylation of H3K18 has been proven to directly stimulate gene expression.^30,32^ Similarly, the lactylation level of H4K5 was significantly enriched in the promoter regions of PD-L1 in cS5-overexpressed AML cells (Fig. 4i). Moreover, by exposing AML cells to a higher concentration of glucose to induce intracellular lactate production, the enrichment of H4K5 lactylation was also increased (Fig. 4j). Hence, lactate-activated PD-L1 gene expression might be through histone lactylation on PD-L1 promoter.

### The nuclear localization of E3BP facilitates H4K5 lactylation in AML cells

**Fig. 5:**
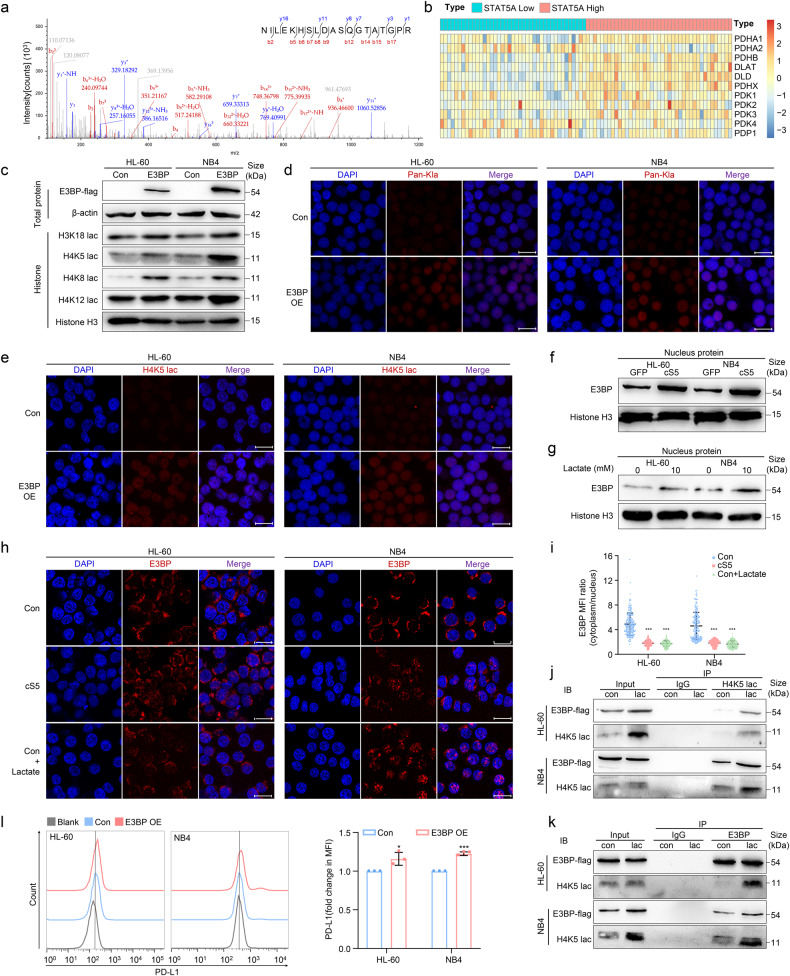
The nuclear localization of E3BP is induced by STAT5 in AML cells**. a** Identification of lactylated-H4K5 interacting proteins were analyzed by LC-MS/MS assay. The peptide spectrum of E3BP was identified and the N-terminal and C-terminal collision-induced dissociation fragment ions were indicated by b and y, respectively. **b** Heatmap visualization of pyruvate dehydrogenase complex in AML patients which were derived from GSE12417 after categorizing into quartiles based on the expression of STAT5A was shown. **c** Site-specific histone lactylation was detected after E3BP overexpression (OE) by western blot. **d**, **e** Equal numbers of control and E3BP-overexpressed AML cells were cultured for 24 h. Histone Kla and H4K5 specific histone lactylation was detected by immunofluorescence. Scale bar = 20 μm. **f**–**h** The localization of E3BP in AML cells was detected by western blot or immunofluorescence. Scale bar = 20 μm. **i** E3BP translocation was quantified by measuring E3BP nuclear foci/distribution in 200 cells. **j**, **k** Nucleoprotein was extracted and incubated with protein A/G magnetic beads preincubated with H4K5 lac or E3BP antibody for Co-IP assay. E3BP or H4K5 lac expression was analyzed by western blot. **l** Equal numbers of control and E3BP-overexpressed AML cells were cultured for 48 h followed by flow cytometry analysis of PD-L1 expression. Data were represented as mean ± SD. *p < 0.05, ***p < 0.001

To search for the molecules that regulated histone lactylation in AML cells, we performed LC-MS/MS to verify the combining proteins of lactylated-H4K5. The result showed that E3BP, the component of pyruvate dehydrogenase (PDH) complex, could interact with lactylated-H4K5 in leukemic cells (Fig. 5a). Meanwhile, AML patients derived from GSE12417 were categorized into quartiles based on the expression of STAT5 followed by an analysis of E3BP expression. The gene expression of E3BP (PDHX) was elevated in STAT5 high-expressed AML patients (Fig. 5b). We next constructed E3BP-overexpressed AML cells followed by detection of histone lactylation. The result showed that E3BP significantly induced histone lactylation, including H3K18, H4K5, H4K8 and H4K12, in AML cells (Fig. 5c). Furthermore, immunofluorescence also revealed that Kla and H4K5 lactylation were increased after E3BP overexpression (Fig. 5d, e). Unexpectedly, E3BP was enriched in nucleus in cS5-overexpressed AML cells, which indicated that E3BP nuclear localization was regulated by STAT5 (Fig. 5f). Consistently, E3BP nuclear translocation was elevated in lactate-treated AML cells (Fig. 5g–i). Besides, Co-IP illustrated that more E3BP interacted with lactylated-H4K5 after lactate treatment (Fig. 5j, k). Consistently, AML cells exhibited higher PD-L1 level after overexpression of E3BP (Fig. 5l). These results illuminated that lactate-promoted E3BP nuclear translocation contributed to histone lactylation.

### STAT5 suppresses T cell activation by upregulating PD-L1 expression

**Fig. 6:**
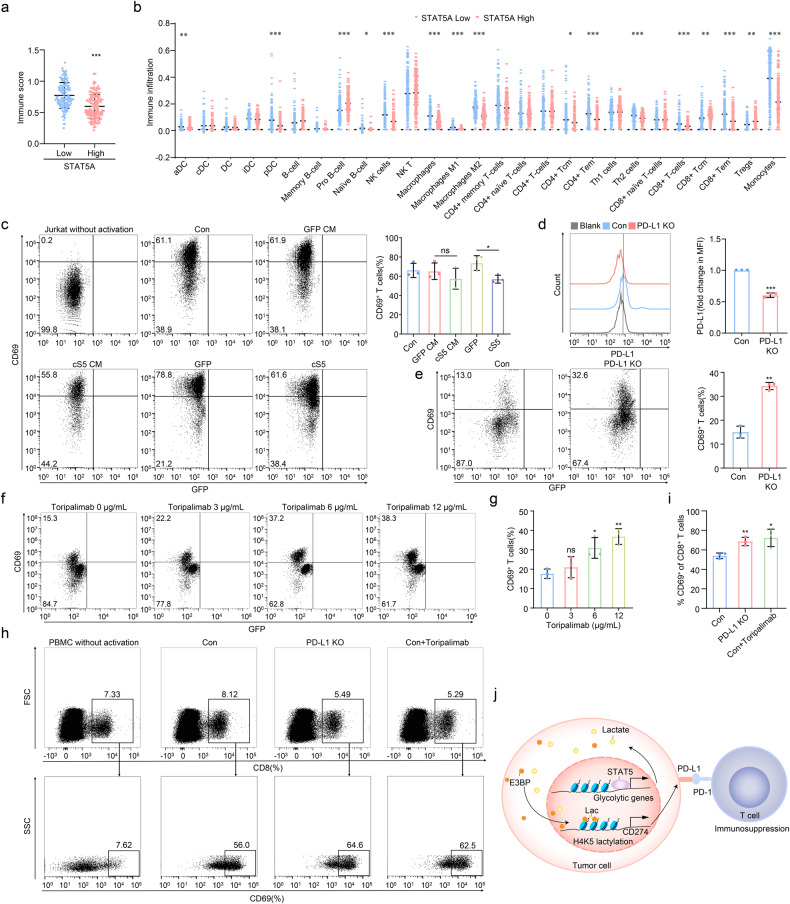
STAT5 upregulates PD-L1 expression followed by T cell suppression. **a**, **b** 524 AML patients derived from GSE14468 were divided by quartiles of STAT5A expression. Immune score and different types of immune cells were analyzed. **c** CM was collected in HL-60 GFP and cS5 after culturing for 24 h. Jurkat cells were co-cultured in CM with or without HL-60 GFP and cS5 cells in 24-well plates accompanied with 2.5 μg/mL anti-human CD3 and 0.5 μg/mL anti-human CD28 stimulation. Cells were then subjected to flow cytometry. **d** PD-L1 knock-out cells were subjected to detection of PD-L1 by flow cytometry. **e** Jurkat cells were co-cultured with HL-60 cS5 (control) or PD-L1 knock-out cells in 96-well plates accompanied with 2.5 μg/mL anti-human CD3 and 0.5 μg/mL anti-human CD28 stimulation. Cells were then subjected to flow cytometry. **f**, **g** Jurkat cells were co-cultured with HL-60 cS5 cells with 0 μg/mL, 3 μg/mL, 6 μg/mL and 12 μg/mL toripalimab in 96-well plates accompanied with 2.5 μg/mL anti-human CD3 and 0.5 μg/mL anti-human CD28 stimulation. Cells were then subjected to flow cytometry. **h**, **i** PBMCs were isolated from AML patients (Supplementary Table S1; No. 25, 26 and 27) and co-cultured with cS5 cells (control) with or without 12 μg/mL toripalimab, and PD-L1 knock-out HL-60 cS5 cells in 96-well plates accompanied with 2.5 μg/mL anti-human CD3 and 0.5 μg/mL anti-human CD28 stimulation. Cells were then subjected to flow cytometry. **j** Model of STAT5 promoting histone lactylation to facilitate PD-L1 expression and driving immunosuppression in AML. Data were represented as mean ± SD. *p < 0.05, **p < 0.01, ***p < 0.001, ns not significant

Based upon our finding that STAT5 drove PD-L1 expression in AML cells (Fig. 3a), we hypothesized that STAT5 might suppress immune function mediated by PD-L1. We characterized the difference in immune landscape between STAT5 high- and low-expressed AML patients in GSE14468. Notably, the immune score was reduced and the content of CD8^+^ T cells was declined in AML blast with high STAT5 expression (Fig. 6a, b). Subsequently, we conducted a co-culture system between AML cells and T cells. There was a comparable activation level of Jurkat cells cultured in conditional medium (CM) derived from cS5 and control AML cells. However, direct contact of Jurkat cells and cS5 AML cells displayed markedly downregulation of CD69 expression in Jurkat cells (Fig. 6c and Supplementary Fig. 4a), suggesting that STAT5 might suppress Jurkat cell activation by inducing PD-L1 expression. We further generated PD-L1 knock-out (KO) HL-60 cS5 cells (Fig. 6d) followed by co-culturing with Jurkat cells. As expected, Jurkat cells were reactivated by PD-L1 knock-out (Fig. 6e). It was noteworthy that knock-out of PD-L1 did not obviously alter the proliferative ability and glucose utilization of AML cells (Supplementary Fig. 4b–d), which implied the activation of Jurkat cells in the co-culture system was mostly due to PD-L1 knock-out. Meanwhile, blockage of PD-1/PD-L1 interaction by treating Jurkat cells with PD-1 neutralizing antibody toripalimab also restored Jurkat cell activation (Fig. 6f, g). To identify the specific type of T cells that responded to PD-1/PD-L1 blockage, a scRNA-seq data of BM cells from relapsed or refractory AML patients who were pre- and post-treated with azacytidine and nivolumab was analyzed. The results showed that CD8 CTL (enriched for cytotoxic markers GZMB, GNLY and PRF1) were significantly increased in treatment-responded AML patients (GSE198052, Supplementary Fig. 4e), suggesting that CD8^+^ T cells could respond to PD-1 treatment in AML. Consistently, CD8^+^ T cells of PBMCs were reactivated after co-culturing with PD-L1-KO HL-60 cS5 cells or exposure to toripalimab (Fig. 6h, i), giving support that PD-L1 inhibition in AML might restore CD8^+^ T cell activation suppressed by STAT5.

## Discussion

Targeting PD-1/PD-L1 has been approved for tumor treatment with durable clinical benefits. Therefore, it is pivotal to explore the molecular mechanism in regulating the expression of PD-1/PD-L1. In this study, we uncovered that STAT5 suppressed CD8^+^ T cell activation by promoting PD-L1 expression in AML. STAT5 promoted glycolysis to accumulate lactate, which facilitated E3BP nuclear translocation, increased the histone lactylation on PD-L1 promoter and subsequently induced PD-L1 transcription. PD-L1 expression in leukemic cells could further attenuate CD8^+^ T cell activation, while blockage of PD-1/PD-L1 interaction reactivated CD8^+^ T cells in STAT5 high-expressed AML. Therefore, STAT5-induced lactate could be as an indicator for the application of immunotherapy to AML.

Immunotherapy makes impressive progress in providing long-term disease control in cancer treatment, including antibody therapy, genetically redirected T cells and immune checkpoint inhibitors.^40^ Of note, checkpoint inhibitors have engaged more attention because of the direct application as clinical compounds. PD-1/PD-L1 engagement has been observed in various cancer types, while the expression of PD-L1 is used to predict the response of patients to anti-PD-1/PD-L1 treatment.^41^ The regulation of PD-L1 has been studied in different aspects, including genomic, epigenetic, transcriptional or post-transcriptional regulation and modification.^42^ It is reported that PD-L1 expression is regulated by oncogenic genes, such as JAK2^V617F^.^43^ Thus, as a direct downstream of JAK2, STAT5 might positively regulate PD-L1 expression. Studies have also revealed that PD-L1 expression can be regulated by tumor-derived metabolites, such as lactate.^38,39^ Consistent with these reports, we revealed that constitutively activated STAT5 promoted glycolysis and induced lactate accumulation, which further increased PD-L1 expression in AML. PD-1/PD-L1 blockage is clinically beneficial mostly by disturbing T cell immune function.^9^ Early study has explored the treatment of ipilimumab in hematologic malignancies in the setting of allogeneic stem cell transplantation disease progression.^15^ In addition, inhibition of PD-1/PD-L1 combined with other targeted therapy has also been proven to improve outcomes in AML patients.^14^ Whereas, there is still a lack of systematic demonstration to apply anti-PD-1/PD-L1 therapy to AML. Therefore, if the indication of immune checkpoint inhibitors is characterized, potential therapeutic benefit would be achieved to AML. Our study showed that blockage of PD-1/PD-L1 could reactivate CD8^+^ T cells when co-cultured with STAT5 constitutively activated leukemic cells, which provided the basis for using immune checkpoint inhibitor in STAT5 high-expressed AML patients.

Lactate-derived lactylation of histone lysine residues serves as a novel type of histone mark.^30^ STAT5 has been implicated in glucose metabolism in various types of cells.^23–25^ For instance, activation of STAT5 increases the expression of GLUT1, GLUT3, GYS2 and UGP2, which subsequently improves glucose uptake in hematopoietic stem cells.^24^ The same as the previous reports, STAT5 increased-lactate accumulation was observed in our study. Furthermore, recent studies reveal that histone lactylation modified by lactate is integrated into tumorigenesis and cancer progression. In ocular melanoma, histone lactylation induces m^6^A reader protein YTHDF2 expression, which recognizes the PER1 and TP53 mRNAs and enhances their degradation, promoting tumorigenesis.^44^ In prostate and lung adenocarcinomas, Numb/Parkin pathway deficiency exerts an increase in lactate production and histone lactylation followed by transcriptional alteration of neuroendocrine-associated genes.^45^ However, whether STAT5 participates in the modification of histone lactylation has not been reported. Interestingly, our results showed that STAT5 promoted lactate production and facilitated histone lactylation, including H3K18, H4K5, H4K8 and H4K12. It has been reported that multiple post-translational modifications can regulate the expression of PD-L1, such as ubiquitination, phosphorylation, glycosylation and palmitoylation,^46^ but the evidence of lactylation contributing to PD-L1 expression is insufficient. We observed that H4K5 lactylation was significantly enriched in the promoter region of PD-L1 to enhance its transcription in STAT5-overexpressed or lactate-treated AML cells. Hence, lactate-activated PD-L1 gene expression was through histone lactylation on PD-L1 promoter.

Up to now, therapeutic strategy that targets PTM has been broadly applied in clinical setting. Although lactylation has been identified with various cellular processes, the specific regulator remains unclear. By using LC-MS/MS, we enclosed that E3BP was involved in histone lactylation, identifying a non-classical role of E3BP in cell metabolism. E3BP is necessary for anchoring dihydrolipoamide dehydrogenase to the dihydrolipoamide transacetylase core of the PDH complexes.^47^ E3BP has also been reported to participate in pathological process. In breast cancer, E3BP acts as an anti-tumor factor since suppression of E3BP by microRNA-27b deregulates cell metabolism and promotes cell proliferation.^48^ Conversely, another study has revealed that E3BP maintains PDH activity and the production of ATP, and knockdown of E3BP inhibits the proliferation of cancer stem cells in esophageal squamous cell carcinoma by regulating CD44 expression,^49^ suggesting that E3BP might exert opposite function in different cancers. In our study, we observed that E3BP was elevated by STAT5 in AML patients. Meanwhile, E3BP might induce PD-L1 expression by facilitating H4K5 lactylation. Thus, E3BP may be identified as a metabolic target in tumor progression. Further study should be performed to confirm whether it could act as a writer of lactylation in AML.

In conclusion, the accumulation of lactate driven by STAT5 facilitated histone lactylation on PD-L1 promoter and ultimately induced PD-L1 expression. Immune checkpoint inhibitor could block the interaction of PD-1/PD-L1 and reactivate CD8^+^ T cells in STAT5 high-expressed AML. Therefore, our study demonstrates a metabolism-epigenetics-immunity network in the AML progression and STAT5-induced lactate may serve as a predictive biomarker for the application of anti-PD- (L)1 immunotherapy in AML.

## Materials And methods

### Cell lines and clinical samples

HL-60 and U937 were purchased from the American Type Culture Collection. NB4 and KG1a were purchased from the Deutsche Sammlung von Mikroorganismen und Zellkulturen GmbH. Cells were maintained in Roswell Park Memorial Institute (RPMI) 1640 medium (Thermo Fisher Scientific) supplemented with fetal bovine serum (FBS, Gibco). 293FT cell was purchased from the Thermo Fisher Scientific and maintained in Dulbecco’s modified Eagle’s medium (Thermo Fisher Scientific), supplemented with 10% FBS.

Peripheral blood (PB) and BM of untreated AML patients or health donors were obtained from The Third Affiliated Hospital of Sun Yat-sen University with the approval of Institute Research Ethics Committee. PBMCs and BMMCs were isolated by ficoll separation.

### Plasmid construction and lentivirus infection

STAT5-overexpressed AML cells carried two mutations (STAT5A^1*6^) were constructed as described previously.^22,50^ E3BP gene was amplified and inserted into pSin-EF2-Puro lentiviral vector by BamHI and EcoRI restriction enzymes. The sequences used for E3BP-flag amplification were: Forward 5′-GTGTCGTGAGGAA TTCATGGCGGCCTCCTGGA-3′, Reverse 5′-AGATGCATGCGGATCCCTACTTGTCATCGTCGTCCTTGTAATCGGCAAGTC-3′. PD-L1 knock-out plasmid was constructed by ligating sgRNA to lenti-CRISPR VII vector. The sgRNA sequences used for PD-L1 knock-out were: 5′-GAACATGAACTGACATGTC-3′, STAT5 knock-down plasmid was constructed by ligating shRNA to PLKO.1-TRC vector. The shRNA sequences used for STAT5 knock-down were: 5′-TCCGGCACATTCTGTACAATG-3′. 293FT cells were co-transfected with E3BP overexpression, STAT5 knock-down or PD-L1 knock-out plasmid and packaging vectors using lipofectamine 2000 transfection reagent (Invitrogen, 11668-019). Lentivirus was collected 48 h after transfection followed by cell infection.

### qPCR assay

The total RNA of each cell type was isolated by TRIzol reagent (Invitrogen, 15596018). cDNA synthesis was performed with Thermo Scientific RevertAid RT Kit (Thermo Fisher Scientific, K1622). qPCR was conducted using Roche LightCycler480 with a ChamQ SYBR qPCR Master Mix (Vazyme, Q311-02). The primer sequences were shown in Supplementary Table S2.

### Immunoblotting assay

Proteins were dispersed by SDS-PAGE and transferred to nitrocellulose membranes (Merck Millipore, HATF00010). The membranes were blocked with 5% BSA at room temperature (RT) for 1 h and subsequently incubated with primary antibodies [STAT5 (Santa Cruz, sc-1081), p-STAT5 (Cell Signaling Technology, CST, 9314 s), HK1 (CST, 2024t), AKT (CST, 4685 s), p-AKT (CST, 4058 s), PFKP (CST, 8164t), PDHA (CST, 3205t), β-actin (Santa Cruz, sc-47778), E3BP (Santa Cruz, sc-377255), Histone H3 (CST, 14269 s), H3K18 lac (PTM Bio, PTM-1406RM), H4K5 lac (PTM Bio, PTM-1409), H4K8 lac (PTM Bio, PTM-1415RM), H4K12 lac (PTM Bio, PTM-1411RM), Pan-Kla (PTM Bio, PTM-1401RM) and Flag-tag (ZenBio, 384091)]. After incubation of secondary antibodies, immunoreactive bands were monitored by the chemiluminescent imaging system (Tanon Science & Technology).

### Histone extraction

Histone protein was extracted following the previously published method.^51^ Briefly, cells were collected and washed with PBS twice and resuspended in 1 mL hypotonic lysis buffer followed by incubating for 30 min on rotator at 4 °C. The intact nucleus was collected after centrifuging at 4 °C. The nucleus was resuspended in 400 µL 0.4 N H_2_SO_4_ overnight and the supernatant was collected. Trichloroacetic acid was added to the final concentration of 33% and incubated on ice for 1 h. Histone was collected by centrifuging and washed with ice-cold acetone twice followed by dissolving in an appropriate volume of ddH_2_O.

### ChIP

For N-ChIP, cells were collected, washed with PBS twice and resuspended in Micrococcal Nuclease (MNase) digestion buffer with protease inhibitor at 37 °C. MNase (Sigma, N3755-50UN) was added for DNA digestion at 37 °C for 5 min, and the reaction was stopped by adding stop buffer to the final concentration of 10 mM Tris, pH7.6, and 10 mM EDTA. The solution was sonicated for 20 s in ice water thrice followed by dialyzing against RIPA buffer at 4 °C for 2 h. After dialysis, the supernatant was collected and incubated with protein A/G magnetic beads (MedChemExpress, HY-K0202) which were preincubated with antibody at 4 °C overnight. Then DNA was collected by HiPure PCR Pure Mini Kit (Magen, D2121-02) after digesting by proteinase K and further used for qPCR. The primers corresponding to PD-L1 promoter region used for ChIP-PCR were shown in Supplementary Table S2.

### Measurement of glucometabolic index

For extracellular glucometabolic index measurement, culture medium was collected at 24 h after treatment with or without various concentrations of glucose or 2-DG. For intracellular glucometabolic index measurement, cS5-overexpressed or STAT5 knock-down AML cells, or cells treated with various concentrations of glucose, 2-DG or specific concentration compounds including 0.01 μM oligomycin, 0.1 μM rotenone, 10 mM lactate, or 0.01 μM antimycin A for 24 h were collected and lysed with RIPA. Then the cell supernatant was collected and stored at −80 °C until used. The glucose (Eton Bioscience, 1200031002), lactate (Eton Bioscience, 1200012002) and pyruvate (Nanjing Jiancheng Bioengineering Institute, A081-1-1) assay kits were used to determine the content of each metabolite following manufacturers’ instruction. The detection of lactate content in the BM was performed by using the Lactate Test Kit (Guangzhou Date Bio-engineering Technology) according to the manufacturer’s instruction.

### Luciferase reporter assay

The promoter of PD-L1, HK1, PFKP or PDHA amplified by ClonExpress MultiS One Step Cloning Kit (Vazyme, C113-02) was inserted into the pGL3-Basic vector. For 293FT cell transfection, cells were co-transfected with pRL-TK, pGL3 empty vector or pGL3 vector containing the promoter of PD-L1, HK1, PFKP or PDHA in 12-well plates with lipofectamine 2000 before indicated treatment. For HL-60 and NB4 transfection, cells were transferred to an electrocuvette (Bio-Rad) with a 0.2-cm electrode gap and mixed with pRL-TK, pGL3 empty vector or pGL3 vector containing the promoter of PD-L1 in Opti-MEM medium (Invitrogen) in a volume of 200 μL. Electroporation was performed with a Bio-Rad Gene Pluser Xcell system. After the pulse, the cells were transferred to RPMI 1640 medium supplemented with 10% FBS. Firefly and Renilla luciferase activities were then measured using the Dual-Luciferase Reporter assay (Promega, E1910) and the ratio of firefly/Renilla luciferase activities was determined.

### Flow cytometry

Cells were collected, washed with PBS twice and resuspended in 100 μL PBS containing primary antibodies including BV421 anti-human CD274 (BD Biosciences, 563738), BB700 anti-human CD4 (BD Biosciences, 566392), BV421 anti-human CD8 (BD Biosciences, 562428), PE/cy anine 7 anti-human CD69 (Biolegend, 310911), FITC anti-human CD233 (Biolegend, 369209), PE/cy anine 7 anti-human CD366 (Biolegend, 345013), APC anti-human CD279 (BD Biosciences, 558694), PerCP anti-human CD45 (BD Biosciences, 347464) for 30 min on ice. For Ki67 analysis, cells were fixed and permeabilized by using eBioscience^TM^ FOXP3/Transcription Factor Staining Buffer Set (Thermo Fisher Scientific, 00-5523-00) and incubated with APC anti-human Ki67 (Biolegend, 350514) for 30 min on ice. Cells were then subjected to flow cytometry analysis by BD FACS Canto instrument. Data were analyzed with Flowjo_V10 software.

### AML cell and T cell co-culture assay

For the experiment of HL-60 GFP and cS5 cells suppressing Jurkat cell activation, AML cells were cultured for 24 h, and the CM was collected after centrifugation. Jurkat cells were transferred to 24-well cell culture plate which was previously coated with 2.5 µg/mL anti-human CD3 (Biolegend, 317326) followed by supplying with 0.5 µg/mL anti-human CD28 (Biolegend, 302934) with/without HL-60 GFP and cS5 cells in 1 mL culture medium for 24 h. For the experiment of STAT5 suppressing Jurkat cell or PBMC activation by lactate-facilitated PD-L1 expression, 96-well cell culture plate was coated with 2.5 µg/mL anti-human CD3 overnight. Subsequently, Jurkat cells or PBMCs were mixed with AML cells in cell culture plate and stimulated by 0.5 µg/mL anti-human CD28 in 200 μL culture medium for 24 h. The activation of Jurkat cells and PBMCs were analyzed by flow cytometry.

### Immunofluorescence staining

Cells with different treatments were fixed in 4% paraformaldehyde and permeabilized with 0.1% Triton X-100. After being blocked with 1% BSA, cells were incubated with E3BP (Santa Cruz, sc-377255), Pan-Kla (PTM Bio, PTM-1401RM) or H4K5 lac (PTM Bio, PTM-1407RM) antibody overnight at 4 °C. Fluorescein-labeled secondary antibodies were added and incubated for 1 h at RT, followed by DAPI staining for nuclei visualization. Images were acquired using a laser confocal microscope (LEICA DMi8).

### Co-Immunoprecipitation

E3BP-flag overexpressed AML cells were cultured with/without 10 mM lactate for 24 h, and then cells were collected for nuclear protein extraction. 40 μL protein A/G beads were incubated with 1.5 μg E3BP (Santa Cruz, sc-377255) or H4K5 lac (PTM Bio, PTM-1409) antibody on a rotor at 4 °C overnight, and then 300 μg nuclear protein was further added. After incubation, protein A/G beads were washed thrice and the protein was analyzed by SDS-PAGE electrophoresis.

### Cell glycolysis stress test assay

Seahorse XF Glycolysis Stress Test Kit (Agilent, 103020-100) was used to test the glycolysis capacity of GFP and cS5-overexpressed AML cells. Briefly, XFe96 Cell Culture Microplates was coated with Cell-Tak (Corning, 354240) and AML cells were cultured in microplates followed by exposure to glucose (10 mM), oligomycin (1 μM) and 2-DG (50 mM) sequentially. The plate was run on Agilent Seahorse XFe96 Analyzer and data was analyzed and plotted using Wave Desktop software.

### Bioinformatics analysis

Untargeted metabolomics analysis and LC-MS/MS were performed by Shanghai luming biological technology co.Ltd (China). GSE116256,^52^ GSE198052,^53^ GSE9476, GSE14468 and GSE12417 datasets were obtained from The Gene Expression Omnibus (GEO, https://www.ncbi.nlm.nih.gov/geo/). Differentially expressed genes were considered as genes with absolute log2 fold change >1.5 and a false discovery rate (FDR) < 0.05 by using the limma package. Gene set enrichment analysis (GSEA) was performed by the clusterProfiler package. Survival analysis was conducted as a Kaplan–Meier curve by using the package of survival and survminer package. Immune infiltration and immune score analysis was performed by the xCell algorithm. For single-cell sequence analysis, the removal of batch effect was performed by harmony package. t-Distributed Stochastic Neighbor Embedding (t-SNE) plots of single cell from AML patients and healthy donors were analyzed by using the package of seurat package. R version 4.1.0 was applied for bioinformatic analysis.

### Statistics analysis

Statistical analysis was performed using GraphPad Prism 8.0 software. Experiments were performed at least in triplicate and all data and error bars were presented as the mean ± standard deviation (SD). Student’s test or Wilcoxon test was used to evaluate statistical significance, defined as *p < 0.05, **p < 0.01, ***p < 0.001. Person’ correlation analysis was used to measure the linear correlation of two variables.

### Supplementary information

Supplementary

## Data Availability

The datasets used in the study are available from the corresponding author on reasonable request.
