# Supplementary material for: STAT5 promotes PD-L1 expression by facilitating histone lactylation to drive immunosuppression in acute myeloid leukemia
Source: Signal Transduct Target Ther. 2023 Sep 30;8:391. doi: 10.1038/s41392-023-01605-2 (PMC10542808; doi:10.1038/s41392-023-01605-2)
Supplement: Supplementary file 1 — Supplementary [file 41392_2023_1605_MOESM1_ESM.pdf]

Supplementary Materials for

STAT5 promotes PD-L1 expression by facilitating histone lactylation to drive immunosuppression in acute myeloid leukemia

Ze-Wei Huang[1], Xue-Ning Zhang[1], Ling Zhang[1], Ling-Ling Liu[1], Jing-Wen Zhang[1], Yu-Xiang Sun[2], Jue-Qiong Xu[1], Quentin Liu[1,3,*], Zi-Jie Long[1,*]

Correspondence to: longzij@mail.sysu.edu.cn, liuq9@mail.sysu.edu.cn

This PDF file includes:

Figures. S1 to S4
Tables. S1 to S2

Figure. S1.

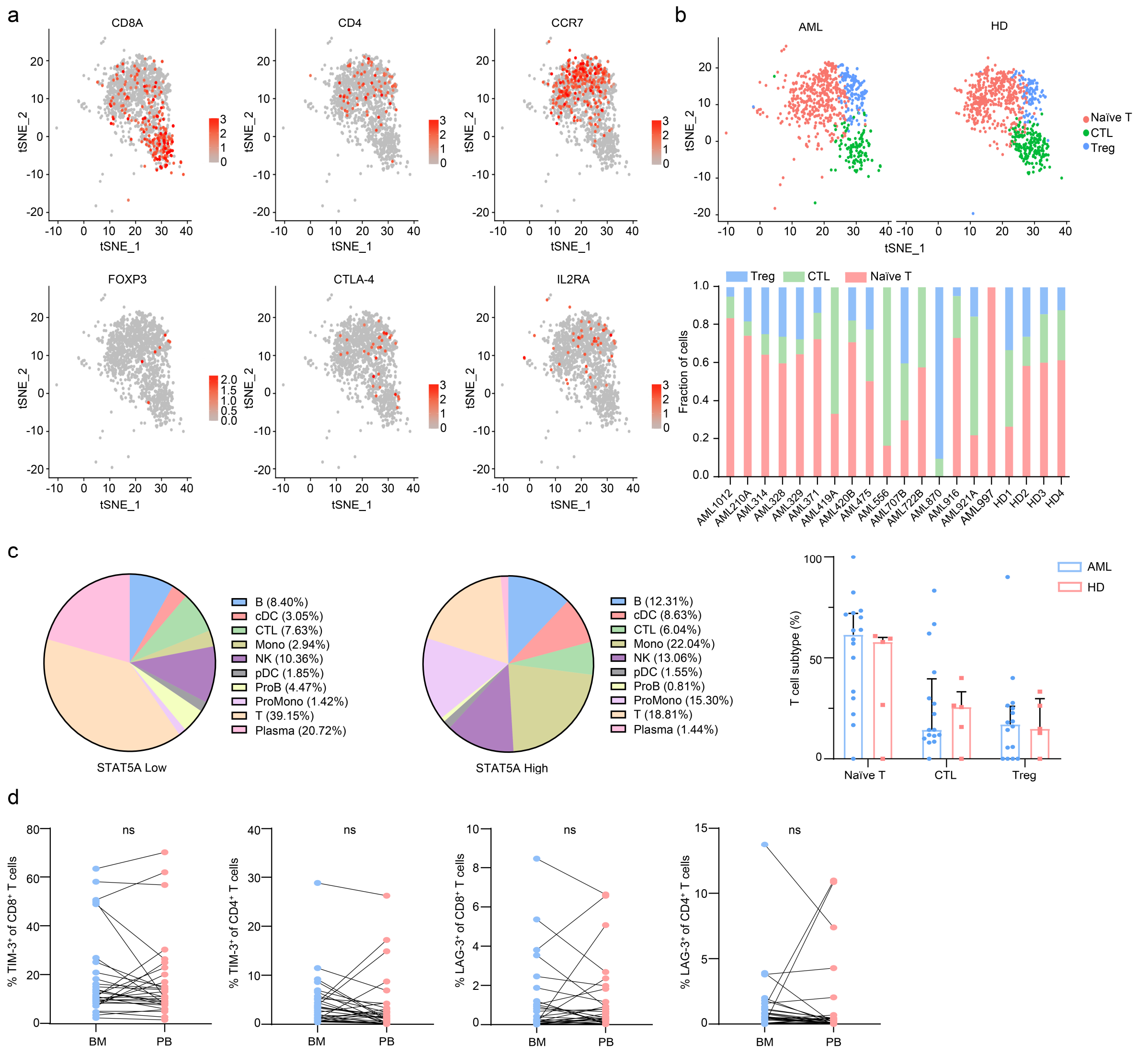

Supplementary Fig. 1 STAT5 dysregulates the function of T cells. a, b T cells from 16 AML patients and 4 healthy donors derived from GSE116256 were classified into CTL, Naïve T, and Treg based on the specific markers on cell surface. T cells were first classified by CD8A and CD4 surface markers, and CTL were defined by CD8A. Subsequently, naïve T cells were identified by CCR7 while regulatory T cells (Treg) were identified by FOXP3, CTLA-4 and IL2RA. The proportion of each type of T cells was numerically displayed and colored. **c** Cells of 16 AML patients derived from GSE116256 were divided by the expression of STAT5A gene. Pie charts showed the relative abundance of immune cells in STAT5A high- and low-expressed AML patients. **d** PBMCs and BMMCs were isolated from AML patients. The expression of TIM-3 and LAG-3 (30 BM vs. 30 PB) was determined. Data were represented as mean ± SD. ns not significant.

Figure. S2.

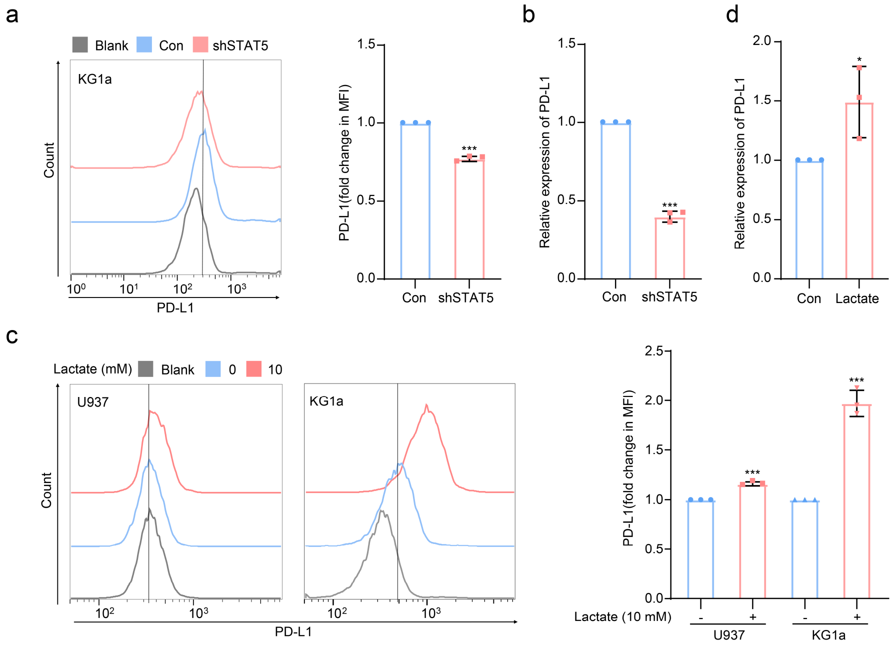

Supplementary Fig. 2 Lactate induces PD-L1 expression in AML cells. a, b Equal numbers of control and STAT5 knock-down KG1a were subjected to flow cytometry and qPCR for PD-L1 expression. **c** U937 and KG1a were exposed to lactate for 24 h respectively and subjected to flow cytometry for PD-L1 expression. **d** AML BMMCs (n=3) were treated with 10 mM lactate for 24 h, and PD-L1 expression was detected by qPCR. Data were represented as mean ± SD. *p < 0.05, ***p < 0.001.

Figure. S3.

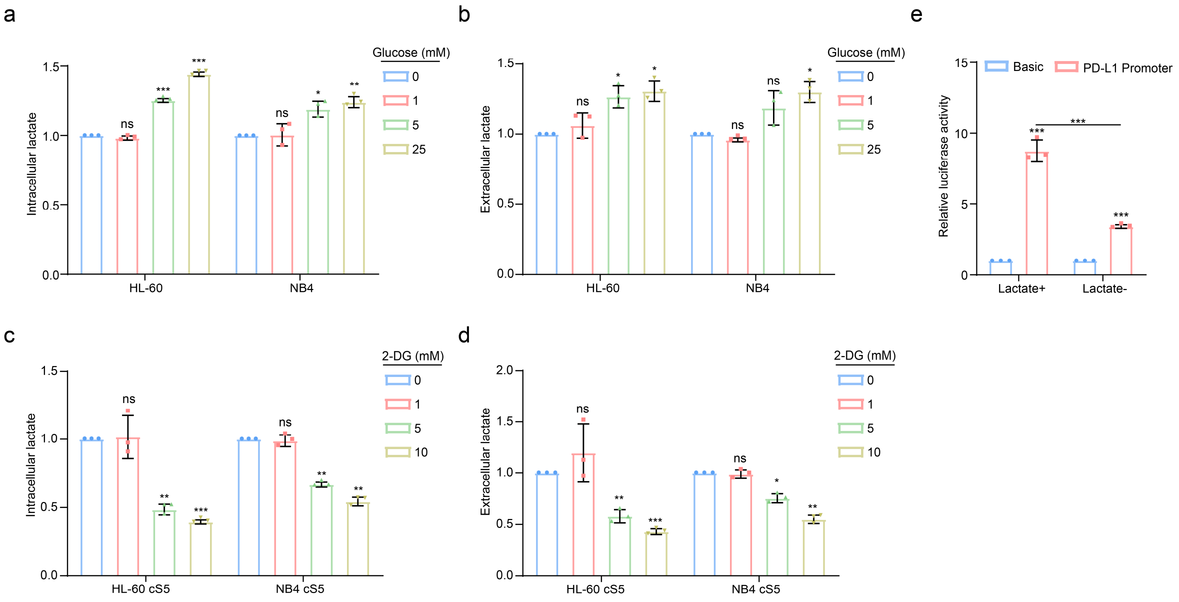

Supplementary Fig. 3 Lactate activates PD-L1 promoter in AML cells. a, b HL-60 and NB4 cells were cultured in glucose-free culture medium and exposed to glucose for 24 h respectively to determinate intracellular and extracellular lactate. **c, d** cS5 AML cells were cultured in glucose-free culture medium supplied with 25 mM glucose followed by exposure to 2-DG for 24 h to determinate intracellular and extracellular lactate. **e** 293FT cells were transiently transfected with pRL-TK, pGL3 basic or pGL3-PD-L1 promoter plasmid followed by luciferase activity determination. For lactate deprivation, the culture medium was changed every 4 h until luciferase activity determination. Data were represented as mean ± SD. *$p < 0.05$, **$p < 0.01$, ***$p < 0.001$, ns not significant.

Figure. S4.

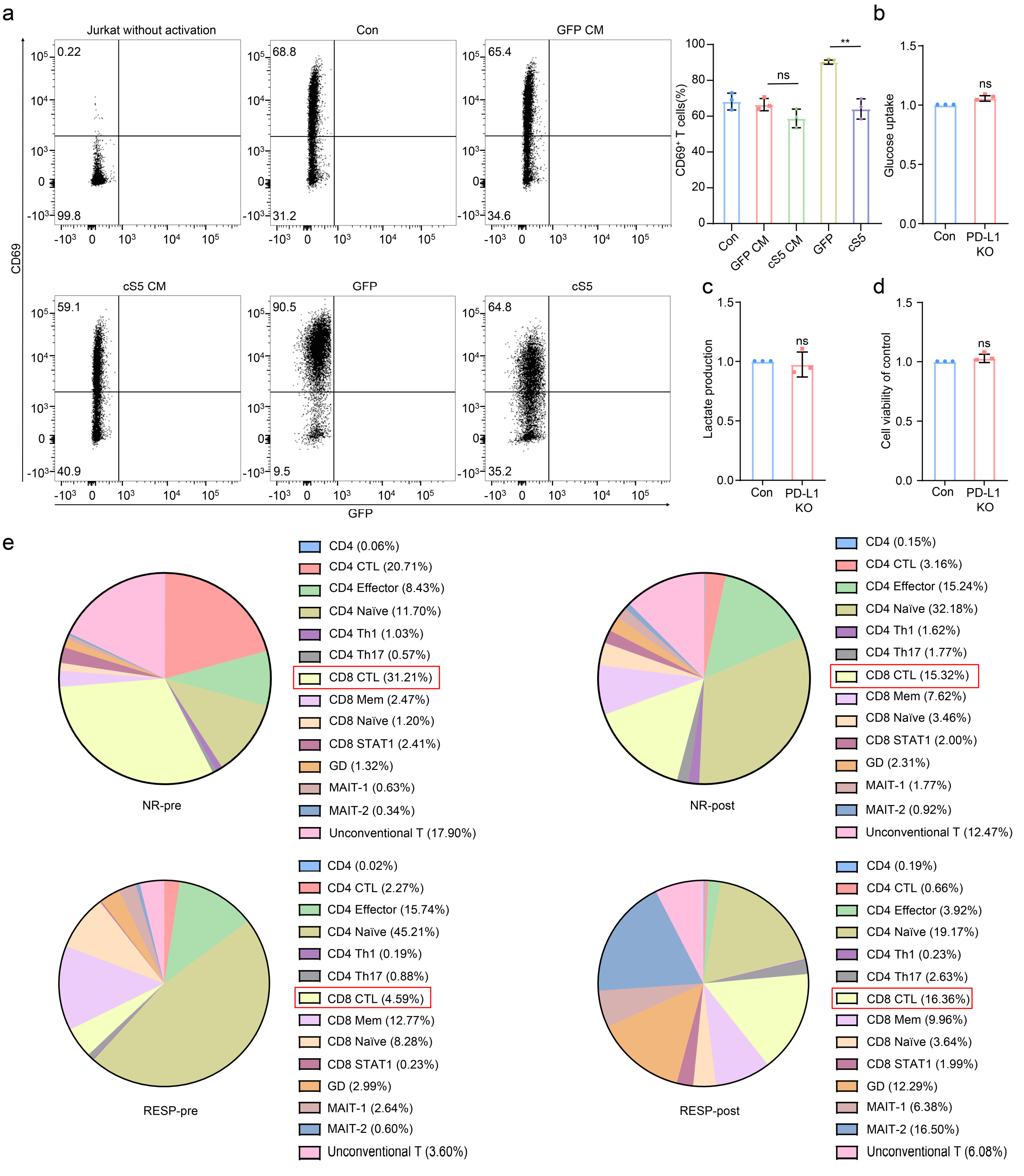

Supplementary Fig. 4 STAT5 represses T cell activation. a CM was collected in NB4 GFP and cS5 cells after culturing for 24 h. Jurkat cells were co-cultured in CM with or without NB4 GFP and cS5 cells in 24-well plates accompanied with 2.5 μg/mL anti-human CD3 and 0.5 μg/mL anti-human CD28 stimulation. Cells were then subjected to flow cytometry. **b-d** Control and PD-L1 knock-out cS5 cells were cultured for 24 h followed by glucose uptake, lactate production and cell viability detection. **e** BM cells of 6 relapsed/refractory AML patients derived from GSE198052 were divided into four groups based on the response to azacytidine+nivolumab

treatment. Pie charts showed the relative abundance of T cell subsets in non-responded (NR) and responded (RESP) AML patients pre- or post-azacytidine+nivolumab treatment. Data were represented as mean ± SD. **$p < 0.01$, ns not significant.

Table S1.

Clinical characteristics of AML patients.

Patient No.	Age(yr)/ Gender	WBC (10^9/L)	FAB	% Blast	Karyotype
1	49/Female	84.82	M1	92.5	-
2	73/Male	7.71	M2	70.5	46,XY
3	35/Female	58.61	M2	79	46,XX
4	31/Female	9.96	M2	79.5	46,XX,t(8;21)(q22;q22)
5	30/Male	82.26	M3	75.5	46,XY,t(15;17)(q24;q21)
6	33/Male	1.0	M3	73	46,XY
7	51/Male	23.32	M3	78	46,XY,t(15;17)(q24;q21)
8	56/Male	14.62	M3	81	46,XY,t(15;17)(q24;q21)
9	38/Male	41.28	M3	88	46,XY,t(15;17)(q24;q21)
10	24/Male	7.9	M5	90.5	-
11	26/Male	11.06	M5	70.5	46,XY
12	50/Female	42.61	M5	89	46,XX
13	41/Male	57.68	M5	86.5	-
14	69/Female	77.1	M5	92.5	46,XX
15	53/Female	163.2	M5	73	46,XX
16	24/Male	55.79	M5	84	46,XY
17	4/Male	94.62	M5	88	-
18	44/Male	61.1	M5	84.5	46,XY
19	48/Male	19.07	M5	71.5	40-42,XY,-5,i(6)(p10),-7,-8,-11, add(12)(p13),-15,inc,-18,+mar1,+mar2
20	57/Male	11.05	M5	75.5	46,XY
21	38/Female	155	M5	92.5	46,XX
22	39/Male	224	M5	93.5	46,XY
23	62/Male	41.11	M5	80	46,XY
24	17/Female	9.98	M5	90	46,XX,t(16;21)(p11;q22)
25	22/Female	21.71	M2	23.5	46,XX,t(8;21)(q22;q22)
26	49/Male	40.18	M2	34.5	46,XY

27	66/Male	0.74	M5	71	46,XY

FAB, French-American-British classification; % Blast, Percentage of leukemic blasts on bone marrow smear; -, Uncategorized.

Table S2.

Primer sequence used for qPCR and plasmid construction.

Gene	Sequence
HPRT-Forward (qPCR)	5’-GCGTCGTGATTAGTGATGATGA-3’
HPRT-Reverse (qPCR)	5’-GCACACAGAGGGCTACAATG-3’
Human STAT5A-Forward (qPCR)	5’-GCAGAGTCCGTGACAGAGG-3’
Human STAT5A-Reverse (qPCR)	5’-CCACAGGTAGGGACAGAGTCT-3’
Mouse STAT5A-Forward (qPCR)	5’-GCAGAAGAAGGCGGAGCA-3’
Mouse STAT5A-Reverse (qPCR)	5’-GGACATGGCGTCAACC-3’
CD274-Forward (qPCR)	5’-TGGCATTTGCTGAACGCATTT-3’
CD274-Reverse (qPCR)	5’-TGCAGCCAGGTCTAATTGTTTT-3’
PDHA-Forward (qPCR)	5’-ATGGAATGGGAACGTCTGTTG-3’
PDHA-Reverse (qPCR)	5’-CCTCTCGGACGCACAGGATA-3’
PFKP-Forward (qPCR)	5’-GCATGGGTATCTACGTGGGG-3’
PFKP-Reverse (qPCR)	5’-CTCTGCGATGTTTGAGCCTC-3’
HK1-Forward (qPCR)	5’-GCTCTCCGATGAAACTCTCATAG-3’
HK1-Reverse (qPCR)	5’-GGACCTTACGAATGTTGGCAA-3’
CD274 promoter-Forward (qPCR)	5’-CAGATGTTGGCTTGTTGTAA-3’
CD274 promoter-Reverse (qPCR)	5’-GTATCTAGTGTTGGTGTCCTA-3’
CD274 promoter-Forward (Plasmid construction)	5’-ATAGGTACCGAGCTCATCTGTTTTGCTTT ACATATTTTTCTG-3’
CD274 promoter-Reverse (Plasmid construction)	5’-GCACGCGTAAGAGCTCTGCCCCCTAGA CCA-3’
PDHA promoter-Forward (Plasmid construction)	5’-ATAGGTACCGAGCTTTTCTATTTTCAT CATTCCTTC-3’
PDHA promoter-Reverse (Plasmid construction)	5’-GCACGCGTAAGAGCTGCTTCTGAGAA GCGC-3’
PFKP promoter-Forward (Plasmid construction)	5’-ATAGGTACCGAGCTAATTGCATAAGG AGATAAGGGGC-3’
PFKP promoter-Reverse (Plasmid construction)	5’-GCACGCGTAAGAGCTCCGTCCGTCCCT CCC-3’

HK1 promoter-Forward (Plasmid construction)	5’-ATAGGTACCGAGCTGAGTTTCACATCT GGCCAGA-3’
HK1 promoter-Reverse (Plasmid construction)	5’-GCACGCGTAAGAGCTGTTCGAGAGCA GCCTGG-3’
